# Hematological, Enzymatic, and Endocrine Response to Intense Exercise in Lidia Breed Cattle During the Roping Bull Bullfighting Celebration

**DOI:** 10.3390/ani15152303

**Published:** 2025-08-06

**Authors:** Julio Sedeño, Salvador Ruiz, Germán Martín, Juan Carlos Gardón

**Affiliations:** 1 Doctoral School, Catholic University of Valencia-San Vicente Mártir, 46001 Valencia, Spain; julio.sedeno@ucv.es; 2 Department of Physiology, Faculty of Veterinary Medicine, Campus Mare Nostrum, University of Murcia, 30100 Murcia, Spain; 3 Institute for Biomedical Research of Murcia, IMIB-Arrixaca, 30120 Murcia, Spain; 4 Department of Basic Sciences, Faculty of Veterinary and Experimental Sciences, Catholic University of Valencia-San Vicente Mártir, 46001 Valencia, Spain; german.martin@ucv.es; 5 Department of Medicine and Animal Surgery, Faculty of Veterinary and Experimental Sciences, Catholic University of Valencia-San Vicente Mártir, 46001 Valencia, Spain

**Keywords:** Lidia breed cattle, blood parameters, exercise, stress, roping bull, animal welfare

## Abstract

The Lidia cattle breed is featured in several traditional popular bullfighting festivals throughout Spain, including the “Toro de Cuerda” event, in which the animals are subjected to intense physical exercise. However, the physiological impact and welfare implications of these activities remain poorly characterized. This study aimed to evaluate the stress response and muscle damage in Lidia breed bulls during roping bull celebrations through comprehensive blood analysis. Blood samples were collected from 53 adult male Lidia bulls before and after a standardized 45 min continuous running exercise during traditional roping bull events in four Spanish autonomous regions. Hematological parameters, muscle enzymes (creatine kinase, lactate dehydrogenase, lactate), and stress hormones (cortisol and ACTH) were analyzed. Significant increases (*p* < 0.05) were observed in leukocytes, lymphocytes, monocytes, eosinophils, neutrophils, erythrocytes, hematocrit, hemoglobin, and post-exercise platelets. Muscle enzymes showed marked elevations, with creatine kinase increasing up to 10-fold above baseline values. Stress hormones, cortisol and ACTH, also demonstrated significant increases. Despite the magnitude of these changes, all parameters remained within established reference ranges for the bovine species. This study provides the first physiological assessment of Lidia cattle during popular bullfighting celebrations, establishing baseline data for evidence-based welfare evaluation and management protocols.

## Simple Summary

Traditional “Roping Bull” celebrations across Spain involve fighting bulls running continuously for 45 min while being chased by participants. Despite their cultural importance, there has been growing public concern about whether these events cause excessive stress or harm to the animals involved. This research aimed to conduct the first comprehensive scientific study to objectively measure the stress experienced by bulls during these popular celebrations, providing factual evidence to inform welfare discussions. We studied 53 bulls from different Spanish regions by collecting blood samples immediately before and after their participation in roping bull events. We analyzed stress indicators in their blood. The bulls showed significant physiological responses, including elevation of stress hormones, white blood cells, and muscle enzymes. Importantly, despite being substantial, all measured changes remained within normal ranges that healthy cattle can safely tolerate. While roping bull events do cause measurable stress to the animals, the bulls’ robust physiology allows them to handle these challenges without exceeding their natural adaptive capacity. This research provides the first scientific foundation for evidence-based animal welfare policies. The findings enable veterinarians, event organizers, regulatory authorities, and the public to make informed decisions about balancing cultural preservation with modern animal welfare standards.

## 1. Introduction

Lidia cattle (*Bos taurus brachiceros*) represent the second most numerous pure breed in Spain’s bovine census, constituting a genetically diverse Iberian population officially recognized under Decree 60/2001 [1,2]. This breed is primarily managed under extensive farming systems across central and southern Spain, with significant populations also present in France, Portugal, Mexico, Colombia, Peru, and other Latin American countries [3,4]. The Lidia breed is distinguished by its characteristic innate aggressive temperament and notable resistance to conventional cattle handling practices, traits that have been genetically selected and preserved over centuries [1,3].

The roping bull (“Toro de Cuerda”) represents one of the most widespread traditional bullfighting celebrations in Spain, involving the subjection of Lidia bulls to situations of intense physical exercise and confrontation with humans. This cultural practice, while deeply rooted in Spanish tradition, has generated increasing concern regarding animal welfare implications [5]. The celebration takes different forms throughout Spain. Each region has its own name for it, such as “bull with rope,” “bou en corda,” “bou capllaçat,” “ensogado,” “enmaromado,” “toro de San Marcos,” or “toro nupcial.” In the Basque Country, it is called “Sokamuturra”. Despite regulatory frameworks governing these events, scientific knowledge regarding their physiological impact on participating animals remains limited and almost unknown, hampering objective welfare assessments [5].

The physiological response to intense exercise in cattle involves complex interactions between metabolic, cardiovascular, and neuroendocrine systems. Previous research has documented various effects of high-intensity exercise in bovines [6,7,8,9,10], while other studies have focused on blood parameter changes following periods of intense physical exertion. Lidia cattle, given their unique behavioral and physiological characteristics, may exhibit particular susceptibility to exercise-induced stress responses. The multifactorial nature of stress during roping bull events encompasses physical stressors (fatigue, tissue injury, pain), environmental factors (handling, transportation, noise), and psychosocial elements (fear, distress) [11,12,13,14,15,16].

In this context, Escalera-Valente et al. [17] conducted an exhaustive study in which they analyzed blood samples from 438 bulls between 4 and 5 years of age, immediately after the bullfight. The results showed significant biochemical alterations, including pronounced elevations in glucose, total protein, and liver and muscle enzymes such as creatine kinase (CK), aspartate aminotransferase (AST), lactate dehydrogenase (LDH), and gamma-glutamyl transferase (GGT). CK values reached extraordinary figures, with some individuals exhibiting levels up to 93,000 IU/L. These levels are well above the established physiological ranges for bovines, indicating severe muscle damage resulting from both physical exertion and the psychological stress to which the animals are subjected.

According to the physiology of exercise, when the energetic demand exceeds the aerobic capacity of the organism, anaerobic metabolism is activated. This leads to an accumulation of lactic acid, a decrease in blood pH, and metabolic acidosis. This disruption of homeostatic balance compromises key metabolic functions and increases the permeability of muscle cell membranes, allowing intracellular enzymes to escape into the bloodstream. In this process, CK is positioned as a particularly sensitive biomarker, given its role in maintaining the energy balance in tissues with a high ATP demand, such as skeletal and cardiac muscle [18]. The findings of Escalera-Valente et al. align with this mechanism, indicating an altered biochemical profile indicative of tissue damage, severe dehydration, and significant mobilization of energy reserves [17].

These findings are consistent with the results previously obtained by Purroy et al. [19], who analyzed 266 male and female fighting bulls after the bullfight. The study revealed that 78% of the animals exhibited histological lesions in skeletal or cardiac muscle, with the majority being chronic. The most frequent alterations included Zenker hyaline degeneration, fibrosis, lymphocytic infiltration, and satellite cell proliferation. These lesions likely remained subclinical until they were exacerbated by intense exercise and acute stress, manifesting in symptoms such as falls or weakness during the fight.

The physiological response of these animals is strongly influenced by the activation of the neuroendocrine stress axes, specifically the hypothalamic–pituitary–adrenal (HPA) axis and the sympathetic–adrenal–medullary system (SAM), which elevates plasma cortisol concentration. Escalera-Valente et al. [17] reported mean values of 117.5 nmol/L, considerably higher than those recorded in other breeds at rest. These results reflect an environment of hyperglycemia, accentuated catabolism, and extreme physiological stress.

Furthermore, studies such as that of Mpakama et al. [18], which focused on slaughter cattle, demonstrated a direct relationship between serum CK levels, the presence of hematomas, and meat quality. The authors emphasize that factors intrinsic to the animal, such as temperament, and extrinsic conditions, such as pre-slaughter handling, can induce muscle damage. Measuring CK in the blood can provide an indication of this type of damage. Elevated CK levels can negatively impact the final quality of the meat product.

The neuroendocrine stress response involves rapid activation of the hypothalamic–pituitary–adrenal axis, resulting in cortisol and ACTH release. These hormones serve as reliable indicators of stress perception and adaptive responses in livestock species. The temporal dynamics of hormone release, with cortisol secretion occurring within 2–3 min of stressor onset, make these parameters particularly suitable for acute stress assessment in field conditions [11,20].

Despite the cultural significance and widespread practice of roping bull celebrations, no previous study has conducted a comprehensive physiological assessment of Lidia cattle during these specific events. The absence of objective, scientifically based welfare data has limited evidence-based discussions regarding the compatibility of these traditions with contemporary animal welfare standards.

The primary objective of this study was to conduct the first detailed assessment of physiological stress responses in Lidia breed bulls during roping bull celebrations through a comprehensive analysis of hematological, biochemical, and hormonal parameters. Specifically, we aimed to (1) quantify acute stress responses through measurement of circulating stress hormones (cortisol, ACTH); (2) identify exercise-induced a possible muscle damage via serum enzyme analysis (CK, LDH, lactate); (3) assess hematological changes indicative of physiological stress; and (4) determine whether observed responses remain within normal physiological ranges for the bovine species. This research provides essential baseline data for evidence-based welfare assessment and management protocol development for traditional bullfighting celebrations.

## 2. Materials and Methods

### 2.1. Ethics

All experimental procedures received approval from the Ethics Committee for Animal Use at the Catholic University of Valencia, San Vicente Mártir (protocol number CEEAUCV2203), conducted following European Union Directive 2010/63/EU for the protection of animals used for scientific purposes and Spanish Royal Decree 53/2013 regulating the protection of animals used for experimentation and other scientific purposes. All activities were conducted under the continuous supervision of the Official Veterinary Services from the respective autonomous communities. The study was designed to minimize animal distress while obtaining scientifically valid data, adhering to the principles of replacement, reduction, and refinement (3Rs) in animal research. All blood sampling procedures were performed by licensed veterinarians experienced in bovine handling to ensure animal welfare and sample quality.

### 2.2. Animals

#### 2.2.1. Study Population

Study animals originated from registered breeding farms in Spain, all officially registered in the Lidia Bovine Breed Stud Book (Libro Genealógico de la Raza Bovina de Lidia, L.G.R.B.L.), maintained by the Spanish Ministry of Agriculture, Fisheries and Food. All farms practiced extensive management systems typical of the Lidia breed, with animals maintained on natural pastures and minimal human intervention before participation in popular celebrations. All participating animals were enrolled in continuous race modality exhibitions officially sanctioned by the Spanish Federation of the roping bull (Federación Española de Toro de Cuerda), ensuring standardized event protocols across different regions.

#### 2.2.2. Inclusion and Exclusion Criteria

Inclusion criteria: (1) Purebred Lidia bovine animals with verified registration in the L.G.R.B.L. and complete individual genealogical identification codes; (2) male animals aged 4 years or older, sexually mature adults in peak physical condition; (3) animals with documented favorable veterinary health certification obtained within 30 days before participation, including tuberculosis and brucellosis testing; (4) clinically healthy animals with body condition score between 3 and 4 on a 5-point scale, indicating optimal nutritional status for athletic performance; (5) Animals with no history of lameness, respiratory disease, or musculoskeletal disorders in the 6 months preceding the study; (6) no pharmacological treatments administered within 7 days before blood sampling to avoid confounding effects on physiological parameters.

Exclusion criteria: (1) Animals showing any signs of illness, injury, or behavioral abnormalities during pre-event veterinary examination; (2) bulls with incomplete genealogical documentation or uncertain breed purity; (3) animals transported more than 6 h immediately before the event to minimize transport-related stress; (4) animals from which adequate blood samples could not be obtained due to technical difficulties or excessive stress during handling.

#### 2.2.3. Sample Size and Geographic Distribution

Fifty-three adult males between 4 and 5 years of age and weighing between 500 and 540 kg of the Lidia breed participated in this study. Sample collection was conducted during the 2023 traditional celebration season across four Spanish autonomous regions: Valencia (*n* = 28, 52.8%), representing the largest sample due to the high frequency of celebrations in this region; Andalusia (*n* = 15, 28.3%); Castilla-La Mancha (*n* = 5, 9.4%); and Aragón (*n* = 5, 9.4%). This geographic distribution ensures representation of different genetic lineages and management practices typical of Spanish Lidia cattle breeding.

### 2.3. Experimental Design and Exercise Protocol

#### 2.3.1. Standardized Exercise Protocol

The roping bull celebration follows a rigorously standardized protocol consisting of continuous running activity lasting exactly 45 min, as regulated by the Spanish Federation of the Roping Bull. Animals were released into predefined rectangular course areas (minimum dimensions: 2000 m × 200 m) and subjected to controlled chase activities by trained participants following traditional celebration rules and safety protocols.

#### 2.3.2. Pre-Event Standardization Procedures

To ensure standardized baseline conditions, all animals underwent identical pre-event management protocols: (1) Minimum 12 h rest period at the event location following transport to allow recovery from transport-related stress; (2) continuous access to fresh water ad libitum throughout the rest period; (3) feed withdrawal for 2 h before initial blood sampling to minimize postprandial effects on metabolic parameters; (4) comprehensive pre-event veterinary examination including assessment of vital signs, locomotion, and general health status; (5) acclimatization period of minimum 30 min in holding areas adjacent to the course to familiarize animals with the environment and reduce novelty stress; (6) standardized handling procedures using experienced personnel to minimize human-induced stress during pre-sampling procedures.

#### 2.3.3. Blood Sampling Protocol

Blood samples were collected at two precisely defined time points to capture the acute physiological response to exercise: (1) Baseline sampling (PRE): collected 2–5 min before commencement of the exercise protocol, during the rest period in designated holding areas, representing true resting physiological status; (2) post-exercise sampling (POST): collected 2–5 min after completion of the 45 min exercise protocol, immediately upon animal restraint to capture peak acute response. The narrow sampling window was specifically selected based on established cortisol release kinetics in cattle and to minimize the confounding effects of prolonged restraint stress on measured parameters. All blood collections were performed by the same team of three experienced veterinarians to ensure consistency in technique and minimize procedural variations.

### 2.4. Sample Collection and Laboratory Processing

#### 2.4.1. Blood Collection Procedures

Blood samples were collected via coccygeal venipuncture using sterile 18-gauge needles (BD Vacutainer^®^ Safety-Lok™ Blood Collection Set, Becton Dickinson, Franklin Lakes, NJ, USA) attached to vacuum tube holders. The collection site was prepared using standard aseptic technique with 70% isopropyl alcohol disinfection. A total of three 5 mL vacuum tubes were collected per animal at each time point: two EDTA K3 anticoagulant tubes (BD Vacutainer^®^ K3 EDTA, 5.4 mg, lavender top) for hematological analysis and hormone determination, and one sodium fluoride/potassium oxalate tube (BD Vacutainer^®^ Fluoride/Oxalate, gray top) for glucose metabolism inhibition and lactate preservation. Collection order was standardized (EDTA tubes first, followed by fluoride tubes) to prevent cross-contamination of anticoagulants. The maximum collection time per animal was limited to 2 min to minimize stress-induced changes in measured parameters.

#### 2.4.2. Sample Processing and Storage Protocols

Immediate processing: EDTA tubes designated for complete blood count analysis were gently inverted 8–10 times immediately after collection and maintained at 4 °C in insulated containers with continuous temperature monitoring. These samples were processed within 2 h of collection to preserve cellular morphology and prevent artifactual changes in cell counts.

Plasma separation: For biochemical and hormonal analyses, EDTA K3 tubes (designated for hormone analysis) and sodium fluoride tubes were centrifuged within 30 min of collection using a refrigerated centrifuge (Eppendorf 5810R, Hamburg, Germany) at 2200× *g* for 10 min at 4 °C, following validated protocols established by Escalera-Valente et al. [17]. Plasma was carefully separated using calibrated pipettes, avoiding the buffy coat layer, and transferred to pre-labeled 1.5 mL polypropylene cryogenic vials (Eppendorf Safe-Lock tubes) with secure caps to prevent sample loss or contamination.

Storage protocol: Plasma samples were initially stored at 4 °C for a maximum of 3 h before transfer to −20 °C freezers for short-term storage. For long-term preservation and to maintain analyte stability, samples were transferred to −80 °C ultra-low temperature freezers within 24 h of collection. Maximum storage duration was 6 months before analysis, with samples thawed only once immediately before analysis to prevent freeze–thaw degradation of labile compounds.

#### 2.4.3. Quality Control Measures

Sample quality was ensured through (1) visual inspection for hemolysis, lipemia, or clotting abnormalities; (2) Temperature monitoring throughout collection and storage using calibrated digital thermometers; (3) chain of custody documentation for all samples; (4) backup sample storage at alternative freezer locations for critical samples; (5) regular equipment calibration and maintenance of all centrifuges and storage equipment.

### 2.5. Sample Analyses

#### 2.5.1. Hematology

Complete blood count (CBC) analysis was performed using a fully automated five-part differential hematology analyzer (Cell-Dyn 3700, Abbott Diagnostics, Abbott Park, IL, USA) specifically calibrated for bovine blood samples using species-appropriate reference standards. The analytical panel included: total white blood cell count (WBC) with automated five-part differential analysis (neutrophils, lymphocytes, monocytes, eosinophils, basophils expressed as percentage of WBC); total red blood cell count (RBC); hematocrit (HCT) measured by impedance methodology; hemoglobin concentration (HGB) determined by spectrophotometric cyanmethemoglobin method; calculated erythrocyte indices including mean corpuscular volume (MCV), mean corpuscular hemoglobin (MCH), and mean corpuscular hemoglobin concentration (MCHC); and platelet count (PLT) with automated size discrimination. All analyses were performed in duplicate with coefficient of variation acceptance criteria of <5% for cell counts and <3% for hemoglobin measurements. Daily quality control was performed using three-level hematology controls (normal, low, high) with results documented in control charts.

#### 2.5.2. Biochemistry

Muscle enzyme analysis: Creatine kinase (CK) activity was measured using the kinetic UV spectrophotometric method based on the coupled enzyme reaction system with hexokinase and glucose-6-phosphate dehydrogenase, monitoring NADPH formation at 340 nm (Biosystems BA400 clinical chemistry analyzer, Biosystems S.A., Barcelona, Spain). The assay was performed at 37 °C with CK-NAC (N-acetylcysteine activated) reagents to ensure optimal enzyme activity. Lactate dehydrogenase (LDH) activity was determined using the standardized pyruvate-to-lactate reaction with simultaneous NADH oxidation monitoring at 340 nm, with results expressed in international units per liter (U/L). Both enzyme assays were calibrated using certified reference materials traceable to international standards, with an inter-assay coefficient of variation maintained below 5%.

Lactate determination: Plasma lactate concentration was measured using an enzymatic method employing lactate oxidase with subsequent colorimetric detection of hydrogen peroxide formation at 505 nm. Samples underwent protein precipitation using perchloric acid before analysis to remove potential interferents and ensure accurate quantification. Results were expressed in millimoles per liter (mmol/L) with an analytical sensitivity of 0.1 mmol/L.

Cortisol analysis: Plasma cortisol concentrations were determined using a fully automated solid-phase competitive chemiluminescent enzyme immunoassay (IMMULITE 1000 Cortisol, Siemens Healthineers, Erlangen, Germany). The assay utilizes a polyclonal rabbit anti-cortisol antibody with minimal cross-reactivity (<0.5%) with other corticosteroids. Analytical sensitivity was 0.2 μg/dL (5.5 nmol/L) with a functional assay range of 0.2–50 μg/dL. Inter-assay coefficient of variation was maintained below 8% across the clinically relevant range. All samples were analyzed in duplicate with an acceptance criterion of <10% coefficient of variation between replicates.

ACTH analysis: Plasma adrenocorticotropic hormone (ACTH) concentrations were measured using a two-site immunoenzymatic sandwich assay (IMMULITE 1000 ACTH, Siemens Healthineers) with analytical sensitivity of 5 pg/mL and functional range of 5–1250 pg/mL. Due to ACTH instability, samples were processed immediately after thawing with aprotinin protease inhibitor (10 μL per mL plasma) added during initial sample collection. The inter-assay coefficient of variation was maintained below 12% throughout the analytical range. Quality control sera were analyzed with each batch to ensure assay performance consistency.

### 2.6. Quality Assurance and Method Validation

Laboratory quality assurance included (1) daily calibration of all analytical instruments using certified reference materials traceable to international standards; (2) duplicate analysis of 15% of randomly selected samples with coefficient of variation acceptance criteria <5% for hematological parameters and <10% for biochemical analyses; (3) participation in external quality assessment schemes for all measured parameters; (4) inter-laboratory comparison testing performed quarterly for hormonal assays; (5) blind quality control samples integrated into analytical runs at 10% frequency; (6) comprehensive documentation of all pre-analytical variables including sample collection time, processing delays, storage conditions, and freeze–thaw cycles; (7) method validation studies performed annually to confirm analytical performance characteristics including precision, accuracy, linearity, and detection limits.

### 2.7. Statistical Analysis

#### 2.7.1. Software and Data Management

Statistical analysis was performed using *R* software version 4.2.3 (R Foundation for Statistical Computing, Vienna, Austria) with additional packages including tidyverse for data manipulation, ggplot2 (4.2.3) for graphical visualization, and psych for descriptive statistics. Raw data were initially assessed for completeness, with missing data patterns evaluated using the VIM package 4.2.3.

#### 2.7.2. Descriptive Statistics and Normality Assessment

Continuous variables are presented as mean ± standard deviation for normally distributed data and median (interquartile range) for non-normally distributed data. Categorical variables are expressed as frequencies and percentages with 95% confidence intervals. Data normality was assessed using the Kolmogorov–Smirnov test for formal statistical testing.

#### 2.7.3. Comparative Analysis Methods

For paired comparisons between PRE and POST measurements, statistical test selection was based on data distribution characteristics determined through normality testing. Normally distributed paired data were analyzed using paired *t*-tests, and non-normally distributed data were analyzed using Wilcoxon signed-rank tests (also known as Mann–Whitney U tests for paired samples). All statistical tests were two-tailed with statistical significance set at *p* < 0.05.

## 3. Results

### 3.1. Hematological Parameters

The reference values of hematological and biochemical variables in cattle, as well as those obtained in the roping bulls evaluated in the present study, are detailed in Table 1 and Table 2.

**Figure 1:**
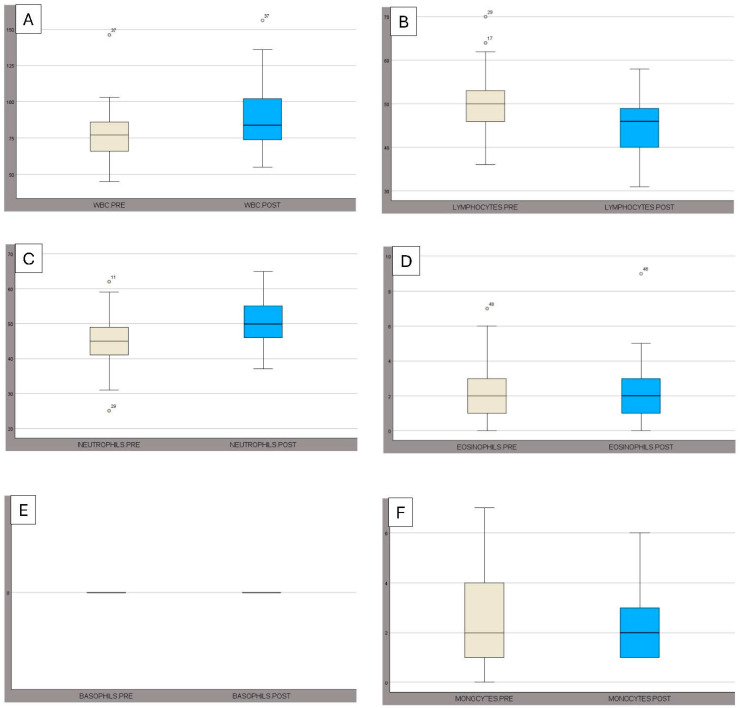
The box plots illustrate the values of (**A**) WBC, (**B**) LYMPHOCYTES, (**C**) NEUTROPHILS, (**D**) EOSINOPHILS, (**E**) BASOPHILS, and (**F**) MONOCYTES, before (PRE) and after (POST) exercise. The boxes extend from the lower quartile to the upper quartile, and the dots indicate the outliers. The error bars represent the standard errors.

**Figure 2:**
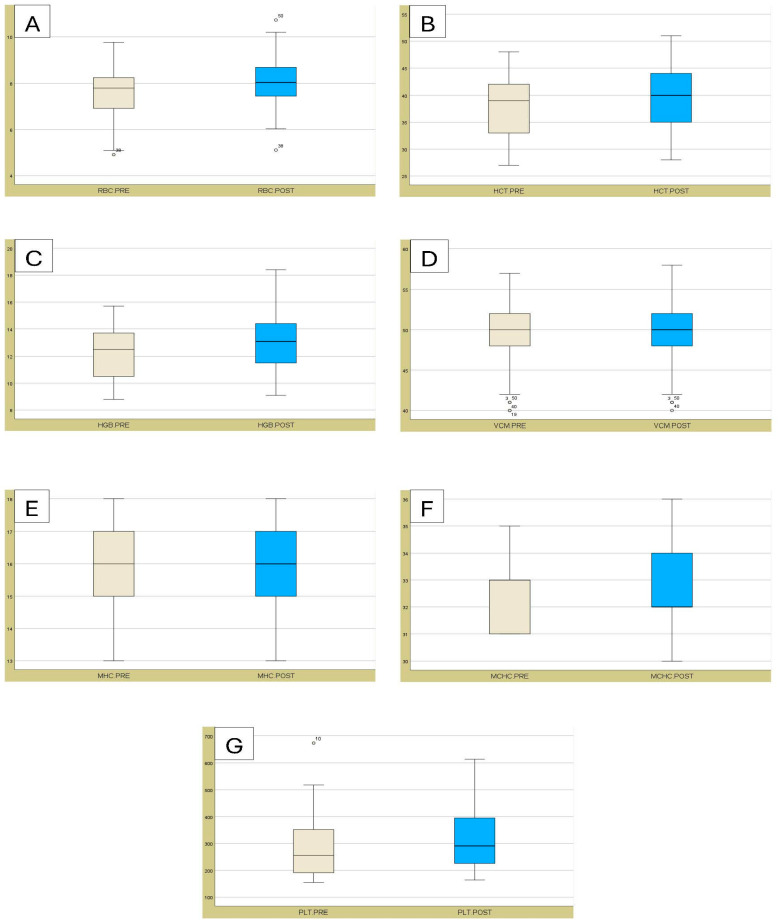
The box plots illustrate the values of (**A**) RBC, (**B**) HCT, (**C**) HGB, (**D**) VCM, (**E**) MHC, (**F**) MCHC, and (**G**) PLT, before (PRE) and after (POST) exercise. The boxes extend from the lower quartile to the upper quartile, and the dots indicate the outliers. The error bars represent the standard errors.

All hematological values exhibited differences between PRE and POST assessments. Significant differences (*p* < 0.05) were observed for WBC, lymphocytes, monocytes, eosinophils, neutrophils, RBC, HCT, HGB, and PLT (Table 1, Figure 1 and Figure 2). Most of the hematological values remained within the normal values for different breeds of cattle, except for neutrophils in POST assessment.

### 3.2. Biochemical Variables

**Figure 3:**
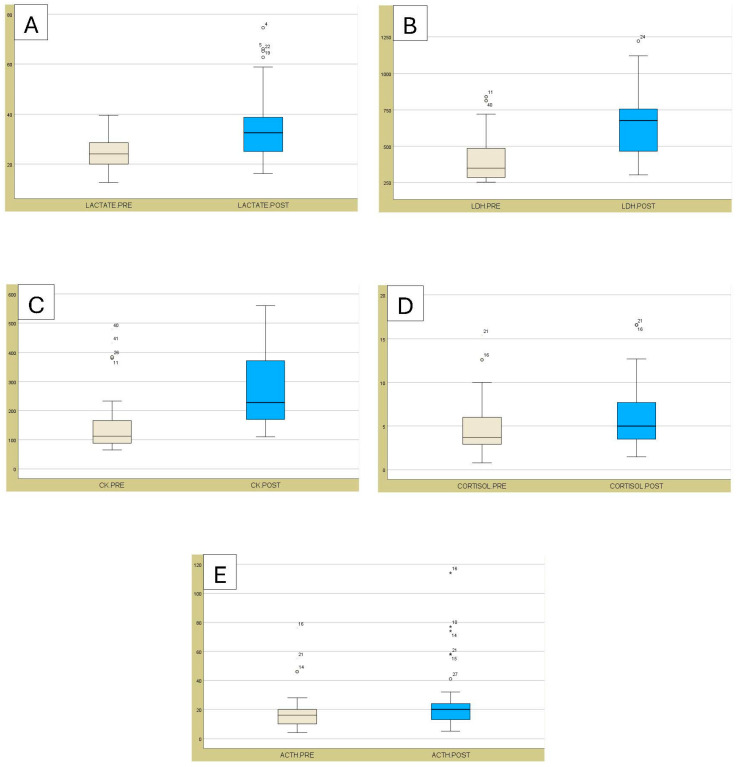
The box plots illustrate the values of (**A**) LACTATE, (**B**) LDH, (**C**) CK, (**D**) CORTISOL, and (**E**) ACTH before (PRE) and after (POST) exercise. The boxes extend from the lower quartile to the upper quartile, and the dots indicate the outliers. The error bars represent the standard errors.

The biochemical variables (enzymes and hormones) obtained from studied bulls are detailed in Table 2 and Figure 3. All biochemical values exhibited marked increases and differed significantly (*p* < 0.001) between PRE and POST assessments. Lactate, LDH, CK, and ACTH values remained within normal values for different breeds of cattle [22]. However, Cortisol values were higher both before (PRE) and after (POST) exercise in comparison to reference values for different cattle breeds (0.5 to 0.76 [21]). Although, our values were similar to those observed for reference values for fighting bulls (1 to 14 [17]).

## 4. Discussion

This study represents the first detailed investigation of hematological and biochemical parameters in Lidia breed animals to determine stress levels during physical activity in roping bull mode, a popular form of bullfighting celebration. The comprehensive physiological assessment provides crucial baseline data for evidence-based animal welfare evaluation in these traditional events.

Among all measured blood enzymes, LDH and CK demonstrated the most significant increases post-exercise. According to the results of the measurements taken after the procedure (POST), the CK activity increased approximately twofold compared to the results of the measurements taken before the procedure (PRE). This magnitude of increase substantially exceeds values typically reported for cattle and approaches levels observed in athletic horses following intense competition [23,24]. The CK elevation serves as direct evidence of exercise-induced muscle membrane damage, reflecting the intense physical demands placed on these animals during the continuous 45 min running protocol.

Comparison with previous research reveals that CK activity levels observed in this study significantly exceeded values reported for cattle exposed to transportation stress, immobilization, or open-field testing [25]. These values were approximately twice those reported by Alonso et al. [17] for fighting bulls under similar exercise conditions, potentially reflecting differences in exercise protocols, animal conditioning, or measurement timing. The observed variability in CK response among individual animals may reflect differences in physical training regimens, which were not systematically implemented during previous decades [26].

The concurrent increases in LDH, CK, and Lactate can be attributed to muscle injury and tissue damage resulting from intense physical activity [12,27,28]. The substantial presence of these enzymes in muscle tissue justifies their utilization as biomarkers of exercise-induced stress. Our findings align with research in other species, where studies in humans have documented elevated LDH, CK, and other enzyme concentrations following marathon and ultramarathon events [29,30]. Similar patterns have been observed in sled dogs undergoing strenuous exercise and in horses following intense training [31,32].

The lactate elevation confirms that the exercise protocol pushed animals beyond their aerobic threshold, necessitating anaerobic metabolism to meet energy demands [10]. Peak Lactate levels of about 78 mg/dL may suggest metabolic stress, which is consistent with high-intensity exercise protocols [10]. The combination of elevated CK and Lactate suggests that the continuous running characteristic of roping bull events represents a significant physiological challenge, leading to both metabolic stress and structural muscle damage [33].

The hormonal response, characterized by marked increases in both cortisol and ACTH, provides unequivocal evidence of hypothalamic–pituitary–adrenal axis activation. Cortisol, secreted within 2–3 min of stressor onset and representing 79–90% of circulating corticosteroids, serves as a primary indicator of stress perception and adaptive response [34,35]. The simultaneous elevation of ACTH confirms the involvement of the central nervous system in the exercise stress response, indicating a genuine activation of the HPA axis and not a peripheral release of cortisol [20].

Stressors present during roping bull events activate both pituitary and adrenal sympathetic systems, resulting in rapid ACTH secretion and subsequent cortisol release. The multifactorial nature of stress in these events—a combination of intense physical exercise, environmental novelty, human proximity, handling, and increased muscle activity in the animal—creates a complex challenge to homeostatic mechanisms [11,16,20]. The hormone levels observed in this study, while elevated compared to baseline, remain within ranges reported for acute stress responses in cattle, suggesting that Lidia bulls retain a capacity for controlled adaptive responses.

Plasma cortisol levels in this study exceeded those typically reported for other cattle breeds under routine management conditions but remained lower than levels documented in some transportation studies [36,37]. The breed-specific stress response characteristics of Lidia cattle, developed through centuries of selection for aggression and environmental resilience, may contribute to their apparent stress tolerance. However, important breed differences in cortisol baseline concentrations have been documented, with some populations showing inherently higher stress hormone levels [38,39].

The hematological changes observed—including increases in total leukocyte count and specific cell populations—represent a classic “stress leukogram” mediated primarily through cortisol-induced leukocyte redistribution [40]. This response pattern, characterized by neutrophilia, lymphocytosis, and monocytosis, differs from the lymphopenia typically associated with chronic stress, confirming the acute nature of the physiological challenge. The concurrent increases in RBC, hematocrit, and hemoglobin likely reflect sympathetic-mediated splenic contraction and hemoconcentration, adaptive mechanisms that enhance oxygen-carrying capacity during periods of increased metabolic demand.

The transportation and handling procedures preceding events may contribute additional stress beyond that imposed by the exercise protocol itself. Transportation can elevate cortisol levels by up to 528% compared to baseline, with normalization typically requiring 24–48 h [41]. While animals in our study had sufficient time for recovery from transportation stress, pre-event handling procedures may have influenced baseline measurements, as suggested by previous research [25].

Physical exercise inherently results in cortisol release through activation of both the sympathetic nervous system and hypothalamic–pituitary–adrenal axis pathways [8]. This phenomenon has been well-documented across species, including horses, where exercise-induced cortisol elevations are routinely observed [42,43]. Breed differences in stress responsiveness have been reported, with some populations showing greater cortisol reactivity than others [38,39]. Temperament variations within breeds also influence stress responses, with more excitable animals typically displaying higher baseline and stimulated cortisol levels [44].

From an animal welfare perspective, these results provide important insights into the physiological cost of roping bull participation. While the observed responses confirm significant stress, the maintenance of all parameters within normal physiological ranges suggests that these events may be compatible with acceptable welfare standards when properly managed. However, several considerations emerge from these findings.

The magnitude of CK elevation indicates substantial muscle damage that requires adequate recovery time. Based on exercise physiology research, CK normalization typically requires 72–96 h following severe exercise-induced damage [45]. This finding suggests the importance of implementing mandatory rest periods between events to allow complete physiological recovery. Additionally, the acute stress response documented through hormone measurements indicates the need to minimize additional stressors in pre- and post-event periods.

Individual animal variability in response magnitude suggests that veterinary assessment and individualized management may be necessary to identify animals at higher risk of exercise complications. The apparent physiological resilience of the breed, evidenced by the maintenance of parameter ranges despite significant stress, should not be interpreted as the absence of welfare problems, but rather as an indication of a robust adaptive capacity of the Lidia breed animal to intense physical exercise.

Future research should focus on characterizing complete recovery profiles following events, comparing different Roping Bull modalities to identify optimal welfare practices, and investigating protective factors that might minimize physiological stress. The development of field-applicable welfare assessment tools and the establishment of evidence-based management protocols would benefit from these foundational physiological data.

These findings contribute essential scientific evidence to ongoing discussions regarding traditional animal use practices and contemporary welfare standards. The results demonstrate that objective physiological assessment can provide valuable guidance for balancing cultural preservation with animal welfare considerations, supporting evidence-based policy development for traditional bullfighting celebrations.

## 5. Conclusions

The intense physical exercise imposed on Lidia breed cattle during roping bull celebrations induces a significant and multifaceted physiological stress response characterized by marked elevations in stress-indicative hematological parameters, substantial increases in muscle damage enzymes (particularly creatine kinase showing 10-fold elevation), and significant activation of the hypothalamic–pituitary–adrenal stress axis, evidenced by elevated cortisol and ACTH concentrations.

While the magnitude of these physiological changes indicates acute stress, most of the measured parameters remained within established reference ranges for the bovine species, suggesting that roping bulls possess adaptive mechanisms capable of managing intense physical challenges. However, the elevation in muscle enzymes, particularly creatine kinase reaching levels comparable to those observed in athletic horses, indicates that these events represent a considerable physiological challenge requiring careful welfare oversight and management.

This study provides the first scientific evidence regarding the physiological impact of Roping Bull celebrations on participating animals, establishing essential baseline data for evidence-based animal welfare assessment. The results support the development of comprehensive management protocols, including mandatory recovery periods between events, pre-participation health assessments, and standardized welfare monitoring procedures to ensure that these important cultural traditions can be maintained within acceptable animal welfare parameters.

## Figures and Tables

**Table 1:** Hematological variables (Mean ± SD) in Roping Bulls (*n* = 53) before performing physical exercise (PRE) and after completion of a continuous 45-min run (POST).

Variable	Unit	PRE	POST	Normality	*p*-Value	Reference Values [21]
WBC	10^3^/µL	7.7 ± 1.8	8.8 ± 2.0	0.085	<0.001	4 to 12
Lymphocytes	%	50.1 ± 6.4	44.7 ± 6.9	0.011	<0.001	45 to 75
Neutrophils	%	44.8 ± 6.5	50.5 ± 6.4	0.066	<0.001	15 to 45
Eosinophils	%	2.4 ± 1.4	2.1 ± 1.6	<0.001	0.019	0 to 20
Monocytes	%	2.5 ± 1.5	2.2 ± 1.3	<0.001	0.039	2 to 7
Basophils	%	0.0 ± 0.0	0.0 ± 0.0	-	-	0 to 2
RBC	10^6^/µL	7.5 ± 1.0	8.0 ± 1.0	0.014	<0.001	5 to 10
HCT	%	37.6 ± 5.5	39.5 ± 5.2	0.012	<0.001	24 to 46
HGB	g/dL	12.1 ± 1.7	12.9 ± 1.7	0.088	<0.001	8 to 15
VCM	fL	49.6 ± 3.9	49.4 ± 3.8	0.020	0.683	40 to 60
MCH	pg	16.1 ± 1.3	16.1 ± 1.2	<0.0001	0.957	11 to 17
MCHC	g/dL	32.4 ± 1.0	32.7 ± 1.5	<0.0001	0.121	30 to 36
PL	10^3^/µL	289.3 ± 113.3	321.6 ± 120.0	0.029	<0.001	100–800

WBC: white blood cell; RBC: red blood cell; HCT: hematocrit; HGB: hemoglobin concentration; MCV: mean corpuscular volume; MCH: mean corpuscular hemoglobin; MCHC: mean corpuscular hemoglobin concentration; PLT: platelets.

**Table 2:** Biochemical variables (Mean ± SD) in roping bulls (*n* = 53) before performing physical exercise (PRE) and after completion of a continuous 45 min run (POST).

Variable	Unit	PRE	POST	Normality	*p*-Value	Reference Values [21,22]
Lactate	mg/dL	24.4 ± 6.1	35.2 ± 13.7	0.004	<0.001	18 to 40
LDH	IU/L	403.6 ± 152.5	661.5 ± 210.8	<0.001	<0.001	250 to 750
CK	IU/L	145.0 ± 89.6	270.2 ± 125.7	<0.001	<0.001	35 to 280
Cortisol	µg/dL	4.6 ± 2.7	6.1 ± 3.4	0.004	<0.001	0.5 to 0.76
ACTH	pg/mL	17.1 ± 12.1	23.9 ± 19.5	<0.001	<0.001	10 to 40

CK: creatine kinase; LDH: lactate dehydrogenase; ACTH: adrenocorticotropic hormone.

## Data Availability

The original contributions presented in the study are included in the article, and further inquiries can be directed to the corresponding author.

## Abbreviations

The following abbreviations are used in this manuscript:

ACTH	Adenocorticotropic hormone or adenocorticotropin
ADP	Adenosine diphosphate
ATP	Adenosine triphosphate
CK	Creatine kinase
HCT	Hematocrit
HGB	Hemoglobin
LBBSB	Lidia Bovine Breed Stud Book
LDH	Lactate dehydrogenase
MCH	Mean corpuscular hemoglobin
MCHC	Mean corpuscular hemoglobin concentration
MCV	Mean corpuscular volume
NAD	Nicotinamide adenine dinucleotide
NADH	Nicotinamide adenine dinucleotide hydrogenase
PLT	Platelets
POST	After
PRE	Before
RBC	Red blood cells
SD	Standard deviation
WBC	White blood cells

## References

[1] Rodríguez Montesinos A. (2002). Prototipos Raciales del Vacuno de Lidia (Breed Prototypes of Fighting Cattle).

[2] (2001). Real Decreto 60/2001, de 26 de Enero, Sobre Prototipo Racial de la Raza Bovina de Lidia. Bol. Of. Estado.

[3] Lomillos J.M., Alonso M.E. (2020). The Lidia Breed: Management and Medicine. Animal Reproduction in Veterinary Medicine.

[4] Lomillos J.M., Alonso M.E., Sánchez García C., Gaudioso V.R. (2012). Evolución del sector de la producción del toro de Lidia en España. Censos y Ganaderías. ITEA-Inf. Tec. Econ. Agrar..

[5] Federación Española de Toro de Cuerda. https://torodecuerda.es/.

[6] Aceña M.C., García-Belenguer S., Gascón M., Purroy A. (1995). Modifications hématologiques et musculaires pendant la corrida chez le taureau de combat. Rev. Med. Vet..

[7] Alonso M.E., Sánchez J.M., Robles R., Zarza A.M., Gaudioso V.R. (1997). Relation entre la fréquence des chutes et différents paramètres hématologiques chez le taureau de combat. Rev. Med. Vet..

[8] Escalera-Valente F., González-Montaña J.R., Alonso de la Varga M.E., Lomillos-Perez J.M., Gaudioso-Lacasa V.R. (2013). Influence of intense exercise on acid-base, blood gas and electrolyte status in bulls. Res. Vet. Sci..

[9] Escribano B., Tunez I., Requena F., Rubio M.D., De Miguel R., Montilla P., Tovar P., Aguera E. (2010). Effects of an aerobic training program on oxidative stress biomarkers in bulls. Vet. Med..

[10] Sánchez J.M., Castro M.J., Alonso M.E., Gaudioso V.R. (1996). Adaptive metabolic responses in females of the fighting breed submitted to different sequences of stress stimuli. Physiol. Behav..

[11] Mota-Rojas D., Napolitano F., Strappini A., Orihuela A., Martínez-Burnes J., Hernández-Ávalos I., Mora-Medina P., Velarde A. (2021). Quality of Death in Fighting Bulls during Bullfights: Neurobiology and Physiological Responses. Animals.

[12] Roches A.d.B.D., Faure M., Lussert A., Herry V., Rainard P., Durand D., Foucras G. (2017). Behavioral and patho-physiological response as possible signs of pain in dairy cows during Escherichia coli mastitis: A pilot study. J. Dairy Sci..

[13] Edwards-Callaway L.N., Cramer M.C., Cadaret C.N., Bigler E.J., Engle T.E., Wagner J.J., Clark D.L. (2021). Impacts of shade on cattle well-being in the beef supply chain. J. Anim. Sci..

[14] García-Torres S., Cabeza de Vaca M., Tejerina D., Romero-Fernández M.P., Ortiz A., Franco D., Sentandreu M.A., Oliván M. (2021). Assessment of stress by serum biomarkers in calves and their relationship to ultimate pH as an indicator of meat quality. Animals.

[15] Kareklas K., Kunc H.P., Arnott G. (2021). Extrinsic stressors modulate resource evaluations: Insights from territoriality under artificial noise. Front. Zool..

[16] Gimsa U., Tuchscherer M., Kanitz E. (2018). Psychosocial stress and immunity—What can we learn from pig studies?. Front. Behav. Neurosci..

[17] Escalera-Valente F., Alonso M.E., Lomillos-Pérez J.M., Gaudioso-Lacasa V.R., Alonso A.J., González-Montaña J.R. (2021). Blood Biochemical Variables Found in Lidia Cattle after Intense Exercise. Animals.

[18] Mpakama T., Chulayo A.Y., Muchenje V. (2014). Bruising in slaughter cattle and its relationship with creatine kinase levels and beef quality as affected by animal related factors. Asian-Australas. J. Anim. Sci..

[19] Purroy A., García-Belenguer S., González J., Gascon M., Barberan M. (1992). Muscular lesions and enzymatic activities in fighting bulls. Ann. Rech. Vet..

[20] Mbiydzenyuy N.E., Qulu L.A. (2024). Stress, hypothalamic-pituitary-adrenal axis, hypothalamic-pituitary-gonadal axis, and aggression. Metab. Brain Dis..

[21] Radostits O.M., Gay C.C., Hinchcliff K.W., Constable P.D. (2006). Veterinary Medicine. A Textbook of the Diseases of Cattle, Horses, Sheep, Pigs and Goats.

[22] Constable P., Hinchcliff K.W., Done S., Gruenberg W. (2017). Veterinary Medicine. A Textbook of the Diseases of Cattle, Horses, Sheep, Pigs and Goats.

[23] Harris R., Marlin D., Dunnett M., Snow D., Hultman E. (1990). Muscle buffering capacity and dipeptide content in the thoroughbred horse, greyhound dog and man. Comp. Biochem. Physiol..

[24] Pösö A.R., Soveri T., Oksanen H.E. (1983). The effect of exercise on blood parameters in standardbred and Finnish-bred horses. Acta Vet. Scand..

[25] Tadich N., Gallo C., Bustamante H., Schwerter M., van Schaik G. (2005). Effects of transport and lairage time on some blood constituents of Friesian-cross steers in Chile. Livest. Prod. Sci..

[26] Grandin T. (1997). Assessment of stress during handling and transport. J. Anim. Sci..

[27] Kramer J.W., Hoffmann W.E., Kaneko J.J., Harvey J.W., Bruss M.L. (2008). Clinical enzymology. Clinical Biochemistry of Domestic Animals.

[28] Brooks G.A. (1986). Lactate production under fully aerobic conditions: The lactate shuttle during rest and exercise. Fed. Proc..

[29] Brancaccio P., Maffulli N., Limongelli F.M. (2007). Creatine kinase monitoring in sport medicine. Br. Med. Bull..

[30] Valberg S.J. (2018). Muscle conditions affecting sport horses. Vet. Clin. N. Am. Equine Pract..

[31] McKenzie E.C., Hinchcliff K.W., Valberg S.J., Williamson K.K., Payton M.E., Davis M.S. (2008). Assessment of alterations in triglyceride and glycogen concentrations in muscle tissue of Alaskan sled dogs during repetitive prolonged exercise. Am. J. Vet. Res..

[32] Arias C., Hernández A., Hernández J. (2013). Plasma biochemistry in Spanish purebred horses: Reference values and evaluation of the effects of training. Comp. Clin. Pathol..

[33] Salamanca Llorente F. (2012). Influencia del Encierro en la Respuesta Fisiológica del toro Durante la Lidia. Ph.D. Thesis.

[34] Chrousos G.P. (2009). Stress and disorders of the stress system. Nat. Rev. Endocrinol..

[35] Sapolsky R.M., Romero L.M., Munck A.U. (2000). How do glucocorticoids influence stress responses? Integrating permissive, suppressive, stimulatory, and preparative actions. Endocr. Rev..

[36] Burdick N.C., Carroll J.A., Hulbert L.E., Dailey J.W., Ballou M.A., Randel R.D., Willard S.T., Vann R.C., Welsh T.H. (2011). Temperament influences endothelial function and inflammation in lactating dairy cows. J. Dairy Sci..

[37] Sporer K.R., Weber P.S., Burton J.L., Earley B., Crowe M.A. (2008). Transportation of young beef bulls alters circulating physiological parameters that may be effective biomarkers of stress. J. Anim. Sci..

[38] Café L.M., Robinson D.L., Ferguson D.M., McIntyre B.L., Geesink G.H., Greenwood P.L. (2011). Cattle temperament: Persistence of assessments and associations with productivity, efficiency, carcass and meat quality traits. J. Anim. Sci..

[39] Curley K.O., Paschal J.C., Welsh T.H., Randel R.D. (2006). Technical note: Exit velocity as a measure of cattle temperament is repeatable and associated with serum concentration of cortisol in Brahman bulls. J. Anim. Sci..

[40] Moberg G.P., Moberg G.P., Mench J.A. (2000). Biological response to stress: Implications for animal welfare. The Biology of Animal Stress.

[41] Earley B., Murray M., Prendiville D.J., Pintado B., Borque C., Canali E. (2012). The effect of transport by road and sea on physiology, immunity and behaviour of beef cattle. Res. Vet. Sci..

[42] McKeever K.H. (2002). The endocrine system and the challenge of exercise. Vet. Clin. N. Am. Equine Pract..

[43] Ferlazzo A., Fazio E., Cravana C., Medica P. (2012). Circulating β-endorphin, adrenocorticotrophic hormone and cortisol levels of horses before and after competitive show jumping with different fence heights. Ann. Anim. Sci..

[44] Cayado P., Muñoz-Escassi B., Domínguez C., Manley W., Olabarri B., Sanchez de la Muela M., Castejon F., Marañón G., Vara E. (2006). Hormone response to training and competition in athletic horses. Equine Vet. J..

[45] Noakes T.D. (1987). Effect of exercise on serum enzyme activities in humans. Sports Med..

